# Impact of recurrent gene duplication on adaptation of plant genomes

**DOI:** 10.1186/1471-2229-14-151

**Published:** 2014-05-31

**Authors:** Iris Fischer, Jacques Dainat, Vincent Ranwez, Sylvain Glémin, Jean-François Dufayard, Nathalie Chantret

**Affiliations:** 1 INRA, UMR 1334 AGAP, 2 Place Pierre Viala, 34060 Montpellier, France; 2 IRD, UMR 232 DIADE, 911 Avenue Agropolis, 34394 Montpellier, France; 3 Montpellier SupAgro, UMR 1334 AGAP, 2 Place Pierre Viala, 34060 Montpellier, France; 4 Université Montpellier II, Institut des Sciences de l'Evolution CC64, Place Eugène Bataillon, 34095 Montpellier, France; 5 CIRAD, UMR 1334 AGAP, Avenue Agropolis, 34398 Montpellier, France; 6 Present Address: Department of Medical Biochemistry, Microbiology, Genomics, Uppsala University, Husargatan 3, 75123 Uppsala, Sweden

**Keywords:** Lineage specific expansion (LSE), Gene duplication, Gene retention, Ultraparalogs (UP), Superorthologs (SO), Comparative genomics, Positive selection, Adaptation

## Abstract

**Background:**

Recurrent gene duplication and retention played an important role in angiosperm genome evolution. It has been hypothesized that these processes contribute significantly to plant adaptation but so far this hypothesis has not been tested at the genome scale.

**Results:**

We studied available sequenced angiosperm genomes to assess the frequency of positive selection footprints in lineage specific expanded (LSE) gene families compared to single-copy genes using a *d*_N_/*d*_S_-based test in a phylogenetic framework. We found 5.38% of alignments in LSE genes with codons under positive selection. In contrast, we found no evidence for codons under positive selection in the single-copy reference set. An analysis at the branch level shows that purifying selection acted more strongly on single-copy genes than on LSE gene clusters. Moreover we detect significantly more branches indicating evolution under positive selection and/or relaxed constraint in LSE genes than in single-copy genes.

**Conclusions:**

In this – to our knowledge –first genome-scale study we provide strong empirical support for the hypothesis that LSE genes fuel adaptation in angiosperms. Our conservative approach for detecting selection footprints as well as our results can be of interest for further studies on (plant) gene family evolution.

## Background

Duplicated genes have been suggested to be the raw material for the evolution of new functions and important players in adaptive evolution [1]. Genomes are constantly subject to rearrangements, by both whole genome duplication (WGD) and small-scale genome duplication (SSD), where tandemly duplicated genes (TDG) are a common case of SSD which generate clusters of physically linked genes. The genomes of angiosperms (flowering plants) are of particular interest to study the impact of gene duplication. Compared to mammals and even to most other plant genomes, angiosperms undergo WGDs, recombination, and retrotransposition more frequently; as a consequence, they also display a larger range of genome sizes and chromosome numbers [2,3]. Most angiosperm genomes sequenced so far show evidence for at least one (but usually more) WGD event during their evolution (see *e.g.*[4-7]). The importance of TDGs has also been shown in *Oryza sativa* (rice) and *Arabidopsis thaliana* where TDGs comprise 15-20% of all coding genes [8-10]. Using genomic and expression data in plants, Hanada *et al.*[11] showed that TDGs tend to be involved in response to environmental stimuli and are enriched in genes up-regulated under biotic stress. This suggests that TDGs play an important role in adaptation of plants to changing environments [11-13]. Taken together, these findings demonstrate the dynamic nature of angiosperm genomes and raise the question of the impact of gene duplications on plant adaptation.

Gene duplication creates an unstable state of functional redundancy, which in most cases will disappear by loss of one copy through accumulation of degenerative mutations, recombination and/or genetic drift. But sometimes both copies are long-term preserved due to functional changes reducing their redundancy and making the loss of one copy disadvantageous [14]. Although the respective roles of adaptive versus non-adaptive processes in the maintenance of gene duplicates have been much debated (for general reviews see [15-18]), gene duplication should increase the occurrence of adaptation for several reasons. First, it can allow the fixation of beneficial mutations on one copy, leading to neofunctionalization, while the other copy ensures the ancestral function [16,19]. Second, it can free the genome from an “adaptive conflict” if the different functions of an ancestral (single) gene cannot be improved independently [20-22]. Third, even when adaptation is not involved in the initial conservation of duplicates, the presence of two (or more) copies is expected to increase the adaptation rate under certain conditions. Duplication increases the number of gene copies, hence the rate of appearance of beneficial mutations. Otto & Whitton [23] showed that if beneficial mutations are dominant or partly dominant, the rate of adaptation should increase with copy number (or ploidy level). If concerted evolution among gene copies is taken into account, Mano & Innan [24] showed that gene conversion (*i.e.* exchange of genetic material between duplicates in a copy and paste manner) increases the effective population size of gene families proportionally to the number of gene members, thus increasing the efficacy of weak selection. Their model predicts that the rate of adaptive substitutions increases with the number of gene copies. Overall we thus expect higher rates of adaptive evolution in multigene families than in single-copy genes.

**Figure 1:**
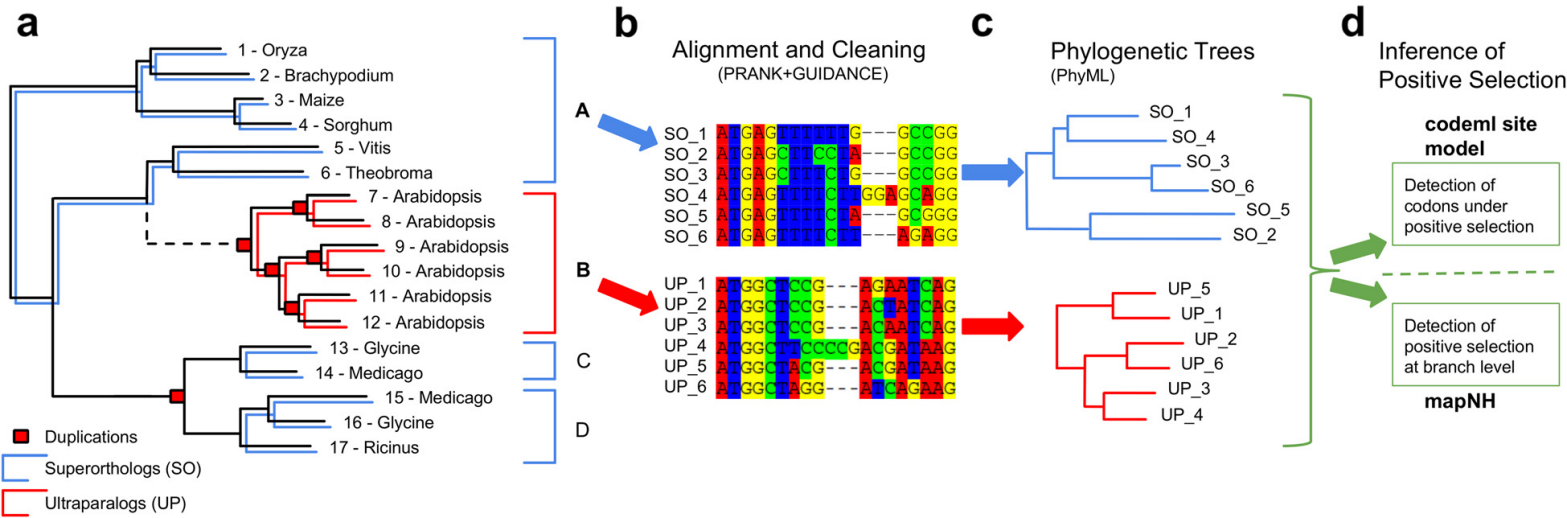
**Workflow overview. (a)** Example of a protein tree as it can be found on the GreenPhylDB. The Arabidopsis sequences 7 – 12 (cluster B) are only related by duplication (nodes with red boxes) and are therefore ultraparalogs (=UP; red lines). Sequences only related by speciation are superorthologs (=SO; blue lines). Those are sequences 1 – 6 (cluster A), 13 and 14 (cluster C), 15 – 17 (cluster D). For example, sequences 13 and 15 are paralogs (as they are related by duplication) but not ultraparalogs (as a speciation event occurred after duplication). We used only clusters containing at least six sequences (clusters A and B, bold) for further analysis. The dashed lines indicate that SO and UP clusters can come from the same GreenPhyl tree (SO1 and UP1 datasets) or from separate trees (SO2 and UP2 datasets). **(b)** Corresponding CDS sequences were downloaded for each cluster. The clusters were aligned using PRANK [71] and cleaned with GUIDANCE [42]. **(c)** For all alignments, phylogenetic trees were created using PhyML [76]. **(d)** Positive selection was inferred on codons using PAML’s codeml [74] and on braches using mapNH [78,79] in all alignments.

As a result of the complex histories of duplicated genes, the retention rate (*i.e.* the proportion of duplicated genes that are maintained in genomes) varies according to several factors including time since the duplication event, protein function, or duplication mode [10]. These variations in retention rates have direct consequences on gene family organization and evolution. Reconciliation methods exploit the observed discrepancies between gene family trees and species trees to infer gene duplication, gene transfer, and gene loss (see [25] for an overview). Among other things, reconciliation methods can be used to estimate duplication or transfer rates and to predict sequence orthology (=sequences related by speciation) [26,27]. Using this method, the extreme heterogeneity of duplication/retention rates among taxa and gene families and/or subfamilies was demonstrated (*e.g.*[28-32]). In particular, reconciliation allows for identification of cases in which recurrent events of duplications (followed by retention) are specific of some lineages and create clades of paralogs (*i.e.* sequences related by duplication) in phylogenetic trees (Figure 1a). Note that since only retained duplications are observable, it is hard to estimate duplication and retention rates independently; hence our use of the “duplication/retention rate” terminology.

Lineage specific duplications/retentions are of particular interest because the recurrence of such events in the same lineage and in a short period of evolutionary time raises the question of their adaptive role to an even greater extent. To test the hypothesis that lineage specific expansion (LSE) of gene families enhances adaptation we compared positive (Darwinian) selection footprints in lineages containing recent and specific duplicated genes to reference lineages containing only single-copy genes. One way to detect positive selection is by analyzing nucleotide substitution patterns at the codon level in a phylogenetic framework. Nucleotide substitutions can either be nonsynonymous (*i.e.* protein changing, thereby potentially impacting the fitness) or synonymous (*i.e.* not protein changing, thereby theoretically without consequences for the fitness). The nonsynonymous/synonymous substitution rate ratio, denoted as *d*_N_/*d*_S_ or ω, can be used to infer the direction and strength of natural selection. If no selection is acting, ω should equal 1. An ω value smaller than 1 indicates an under-representation of nonsynonymous substitutions, which can be interpreted as the preferential elimination of deleterious mutations by purifying selection. The closer ω is to zero, the stronger purifying selection is acting. On the other hand, if ω is larger than 1 it indicates an over-representation of nonsynonymous substitutions, which can be interpreted as positive selection on new variants. Using such an approach, positive selection has been detected for MADS-box transcription factors [33], monosaccharide transporters [34], genes involved in a triterpene pathway [35], an anthocyanin pathway enzyme encoding gene [36], and epimerase genes [37] to mention only a few examples in plants. So far, this approach has mostly been applied to single candidate gene families. Thanks to the availability of numerous completely sequenced plant genomes, it can now be used at the genome level for several angiosperm species.

The dynamic nature of angiosperm genomes makes them an ideal system to study the link between gene duplication/retention rate heterogeneity and adaptation. Assuming that adaptation is acting when positive selection footprints are detected, we want to test if positive selection can be observed more frequently in LSE genes compared to single-copy genes. We applied a *d*_N_/*d*_S_-based test to detect positive selection as it is easy to use on a large scale, it is one of the most stringent tests [38-40], and it has been applied successfully in many similar cases (for examples, see above). Using this approach, we found 5.38% of codons under positive selection in LSE gene families but none in single-copy ones. In addition, the average ω over branches of LSE gene trees is almost twice as high as that observed in single-copy gene trees. We also found a much higher proportion of branches under positive selection and/or relaxed constraint among LSE gene trees than among single-copy gene trees. Taken together, these results strongly support the prediction that (at least in angiosperm genomes) LSE gene evolution plays an important role in adaptation whereas very few single-copy genes seem to be involved.

## Results

### Dataset description

We investigated whole genomes of five monocots (*Musa acuminata*, *O. sativa*, *Brachypodium distachyon*, *Zea mays*, *Sorghum bicolor*) and five dicots (*Vitis vinifera*, *A. thaliana*, *Populus trichocarpa*, *Glycine max*, *Medicago truncatula*). From the GreenPhyl database [41] we extracted ultraparalog clusters (UP – sequences only related by duplication) which represent our LSE gene set. As a single-copy gene reference, we chose a superortholog gene set (SO – sequences only related by speciation). To address the question of whether or not positive selection is more frequent during LSE events, we compared the results obtained on UPs with those obtained on SO gene sets. The SO gene set was then divided in two subsets. The first one, SO1, contains SO genes extracted from GreenPhyl protein trees in which at least one UP cluster was also identified. This means that all the trees from which an SO1 was extracted contain at least one UP cluster. The second SO set (SO2) is the complement of SO1, *i.e.* it is composed of SO genes extracted from GreenPhyl trees in which no UP clusters were found. Likewise, the UP1 dataset represents UP clusters extracted from GreenPhyl trees also containing SO clusters and the UP2 dataset represents UP clusters from GreenPhyl trees from which no SO clusters were extracted. We subdivided the dataset as we expected a “family effect”. This effect may be caused by an accelerated evolutionary rate in some families which are more prone to gene duplication and/or retention than others, *e.g.* due to their function or base composition. If one GreenPhyl tree contained more than one SO or UP cluster, we kept only one cluster randomly (see Methods for details). A detailed overview of the workflow can be found in Figure 1.

**Figure 2:**
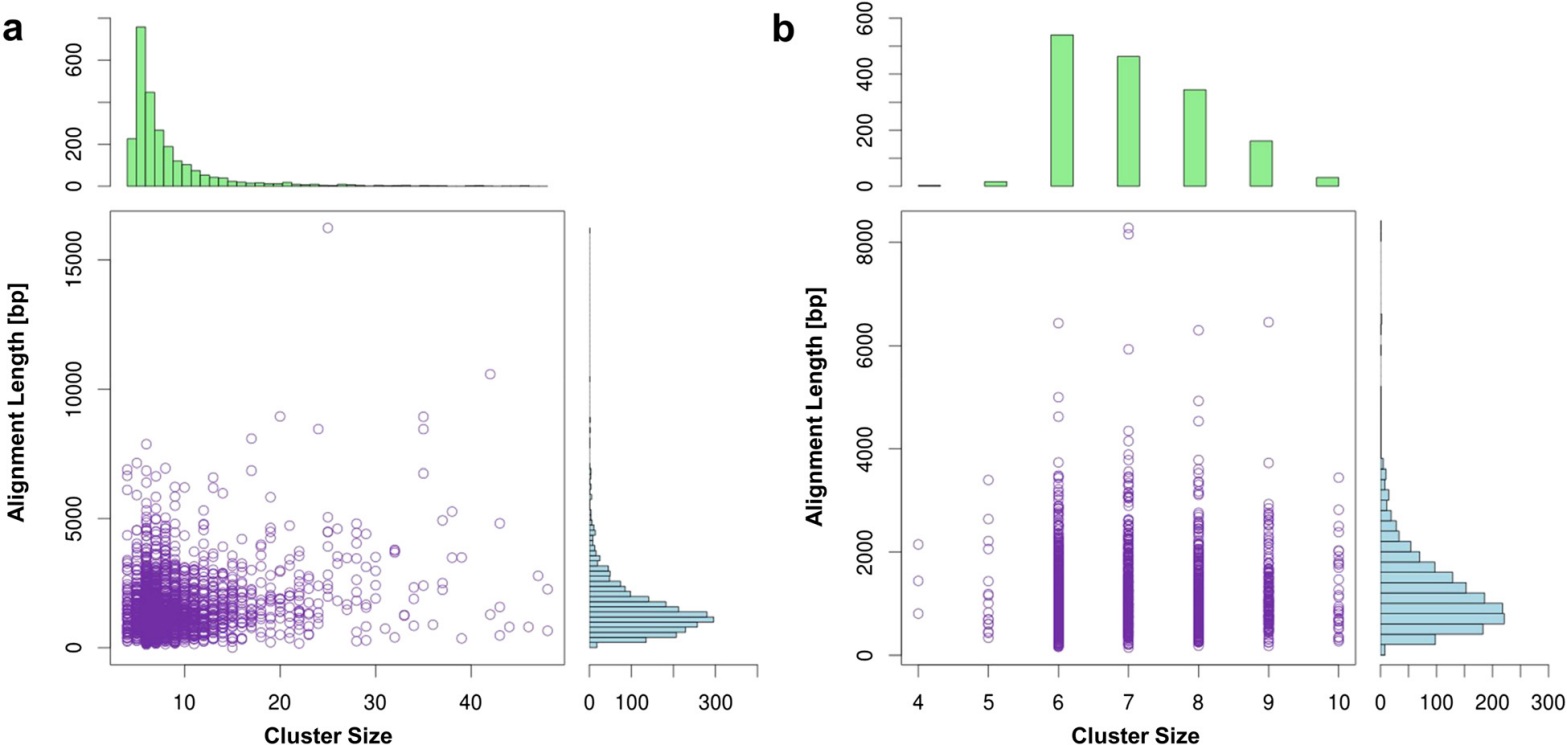
**Alignment length against cluster size.** Each dot in the scatter plot represents **(a)** an ultraparalog or **(b)** a superortholog alignment. The histogram above the scatter plot represents the count of alignments for each cluster size; the histogram right to the scatter plot represents the counts of alignments for each alignment length. bp: base pairs.

Our final dataset for codeml analysis comprised 160 UP1, 1,512 UP2, 167 SO1, and 1,203 SO2 clusters (Table 1). The mapNH analysis was performed on 154 UP1, 1,435 UP2, 167 SO1, and 1,203 SO2 clusters (Table 1) and 1,257 UP1, 14,326 UP2, 1,807 SO1, and 13,374 SO2 branches (Table 1). The median length of the UP1 alignments is 1,272 bp (base pairs), 1,220 bp for the UP2, 1,230 bp for SO1, and 987 bp for SO2 alignments (Table 1, Figure 2). The UP alignments are significantly longer than the SO alignments (Mann–Whitney test: *p* < 0.001). This can be partially explained by the fact that GUIDANCE introduces gaps instead of aligning ambiguous sites [42]. Therefore, UP genes – which are frequently under less selective constraint – may produce longer alignments due to the introduction of gaps. The median number of sequences in an alignment (*i.e.* median cluster size) is 7 for UP and SO alignments (Table 1, Figure 2). We found that the cluster sizes for the SO datasets are significantly smaller than for the UP datasets (Mann–Whitney test: *p* < 0.001) which was expected because the number of sequences a superortholog cluster can contain is at most ten (=number of species used in this study) whereas it is not bounded for UP clusters.

**Table 1:** General dataset description

	**UP1**	**UP2**	**UPps**	**SO1**	**SO2**
Clusters for final codeml site model analysis	160	1,512	90	167	1,203
Clusters for final mapNH analysis	154	1,435	90	167	1,203
Total number of branches	1,881	22,475	1,730	1,817	13,537
Number of analysed branches by mapNH	1,257	14,326	1,298	1,807	13,374
Median cluster size (1^st^ Qu; 3^rd^ Qu)	7 (6; 8)	7 (6; 10)	8 (6; 13)	7 (6; 8)	7 (6; 8)
Median alignment length (1^st^ Qu; 3^rd^ Qu) [bp]	1,272 (792; 1,858)	1,220 (753; 1,851)	1,314 (864; 1,942)	1,230 (900; 1,737)	987 (651; 1,470)
Total number of branches	1,881	22,475	1,730	1,817	13,537
Total number of sites	42,706	355,486	21,864	59,191	340,556

*Qu* quantile; *bp* base pairs.

**Figure 3:**
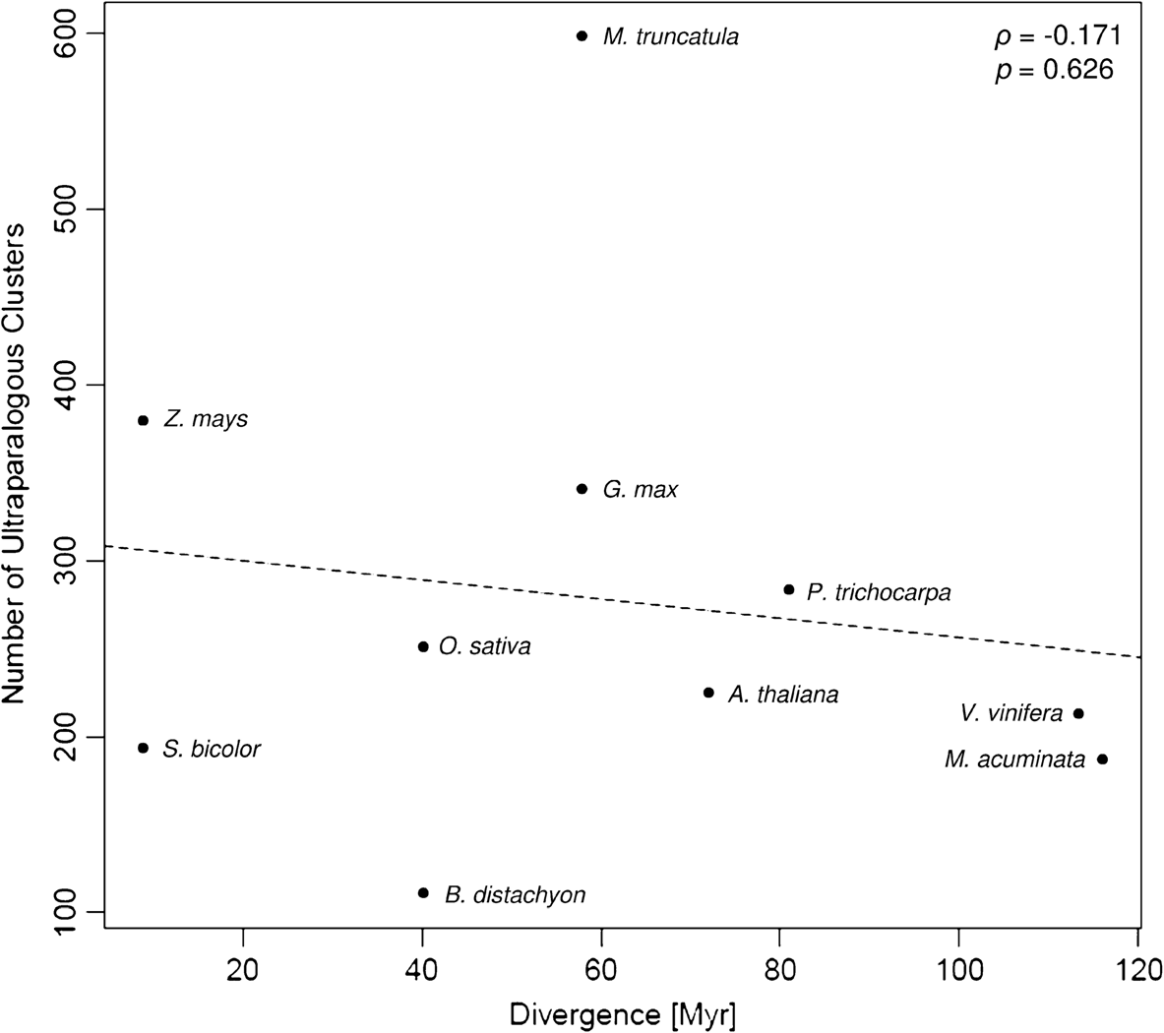
**Number of detected UP clusters for every species against divergence time.** No significant correlation was observed (ρ: −0.171, *p* = 0.626).

As this divergence time between one species and its closest relative increases, one might expect that the number of detected UPs could also increase when compared to a distantly related species than to a closely related one. Therefore, we tested if the divergence time and the number of identified UP clusters correlated. Note that we always used the divergence time relative to the most closely related species in the GreenPhyl database, no matter if we analysed this species later (divergence times can be found in Additional file 1: Figure S1). Regression analysis shows that there is no significant positive correlation between the divergence time and the number of detected clusters: Spearman non-parametric correlation coefficient (ρ) = −0.171, *p* = 0.626 (Figure 3). The correlation remains not significant after removing *M. trunculata* (*ρ* = −0.227, *p* = 0.557). The most likely explanation for this lack of correlation is the equilibrium between gene duplication and loss over time. The birth/death rate has been shown to be relatively constant over time and therefore the frequency of gene copies in a genome declines exponentially with age [14].

### Positive selection at the codon level

The average number of UP clusters used in the final analysis is around 150 clusters per species, with *Brachypodium distachyon* showing a very low (63) and *Medicago truncatula* showing a very high (400) number of clusters (Table 2). On average, 12.86% and 5.38% of UP clusters show evidence for positive selection before and after manual curation, respectively (Table 2). This discrepancy shows how important manual curation for alignment errors is as we discovered around 50% of alignments with a possible false positive signal. As we were very strict during the manual curation process, the clusters remaining can be considered as true positives but we might have removed some other true positives. There is no significant difference between the number of UP1 and UP2 clusters under selection although we detected less – sometimes zero – clusters with codons under selection in the UP1 dataset, most likely because of a small sample size in this dataset (160 clusters vs. 1,512 UP2 clusters). Interestingly, no SO1 or SO2 cluster seems to have evolved under positive selection (Table 2). We also defined a new sub-category of clusters denoted UPps that contains the 90 UP clusters for which positive selected sites were detected and manually validated (Table 1). The UPps clusters have a longer median length (1,314 bp) and larger median cluster size (8) than the other UP and SO clusters (Table 1).

**Table 2:** Clusters containing codons under positive selection before and after manual curation

	**Clusters used in final analysis**		**Clusters under selection before manual curation (%)**		**Clusters under selection after manual curation (%)**	
**Species**	**UP1**	**UP2**	**UP1**	**UP2**	**UP1**	**UP2**
*M. acuminata*	36	107	1 (2.78)	6 (5.61)	0 (0.00)	4 (3.74)
*O. sativa*	7	145	1 (14.29)	29 (20.00)	1 (14.29)	11 (7.59)
*B. distachyon*	4	59	0 (0.00)	14 (23.73)	0 (0.00)	2 (3.39)
*Z. mays*	24	226	4 (16.67)	32 (14.16)	0 (0.00)	9 (3.98)
*S. bicolor*	4	93	0 (0.00)	9 (9.68)	0 (0.00)	4 (4.30)
*V. vinifera*	9	114	1 (11.11)	10 (8.77)	0 (0.00)	3 (2.63)
*A. thaliana*	13	138	0 (0.00)	25 (18.12)	0 (0.00)	14 (10.14)
*P. trichocarpa*	16	132	3 (18.75)	18 (13.64)	1 (6.25)	12 (9.09)
*G. max*	17	128	3 (17.65)	5 (3.91)	3 (17.65)	1 (0.78)
*M. truncatula*	30	370	5 (16.67)	49 (13.24)	4 (13.33)	21 (5.68)
*Sum/average*	*160*	*1,512*	*18 (11.25)*	*197 (13.03)*	*9 (5.63)*	*81 (5.36)*
*UPall*	*1,672*		*215 (12.86)*		*90 (5.38)*	
SO1	167		1 (0.60)		0 (0.00)	
SO2	1,203		3 (0.25)		0 (0.00)	

### ω at the branch level

**Figure 4:**
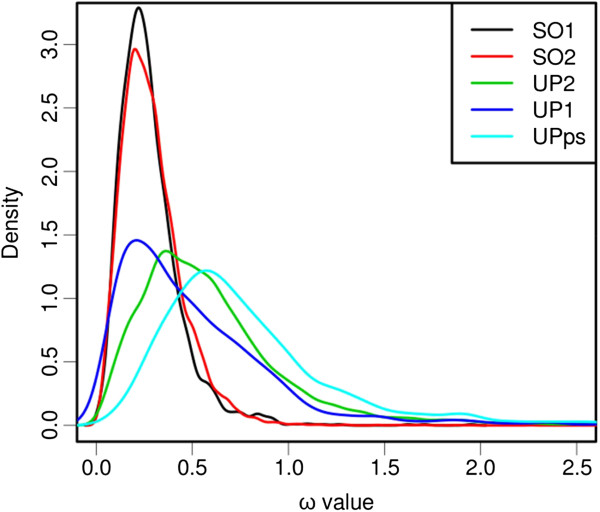
**Distribution of ω of branches in different subsets.** Distribution of ω of branches in SO1 (black), SO2 (red), UP1 (green), UP2 (dark blue), and UPps (light blue) clusters.

The analysis of selective pressures at the branch level was performed using mapNH on the same dataset as the codon analysis. If ω at a branch is larger than 1.2 we consider this a strong indicator of positive selection (simply defining ω > 1 as an indicator of positive selection might lead to false positives as in a neutral scenario ω rather fluctuates around 1 than being exactly 1). The mean ω of the branches is significantly (*p* < 0.001) higher in UP2 (0.62) than in SO2 (0.29) and the distribution shows a larger variance for UP2 than for SO2 (Figure 4, Table 3). As compared to SO2, in UP2 we observe: (i) a higher proportion of branches with ω > 1.2 (8.78%, compared to 0.22% for SO2), (ii) higher ω values for branches with ω > 1.2 (1.80, compared to 1.64 for SO2), and (iii) higher ω values for branches with ω < 1 (0.49 compared to 0.29 for SO2; Table 3). This indicates a relaxation of purifying selection for UP2 in contrast to SO2 but also a higher frequency of branches harboring an accelerated evolution rate. Similar results are observed on the UP and SO clusters extracted from the same trees (*i.e.* UP1 and SO1). Mean ω is significantly (*p* < 0.001) higher for UP1 (0.51) than for SO1 (0.28; Table 3). Interestingly, the mean ω for UP1 and UP2 differ significantly (*p* < 0.001; Table 3, Figure 4), indicating the family effect mentioned before. For the UPps clusters, the mean ω (0.84), the proportion of branches with ω > 1.2 (15.79%), and the mean ω of branches with ω > 1.2 (1.95) are higher compared to the UP1 and UP2 clusters (Table 3, Figure 4).

**Table 3:** Results of the branch analysis with mapNH

	**UP1**	**UP2**	**UPps**	**SO1**	**SO2**
Number of analysed branches	1,257	14,326	1,298	1,807	13,374
Branches with ω < 1 (%)^a^	1,144 (91.01)	12,515 (87.36)	993 (76.50)	1,799 (99.56)	13,329 (99.66)
Mean ω for branches with ω < 1	0.41	0.49	0.59	0.28	0.29
Branches with ω > 1 (%)^a^	113 (8.99)	1,811 (12.64)	305 (23.50)	8 (0.44)	45 (0.34)
Mean ω for branches with ω > 1	1.55	1.52	1.67	1.37	1.44
Branches with ω > 1.2 (%)^a^	73 (5.81)	1,099 (8.78)	205 (15.79)	4 (0.22)	23 (0.17)
Mean ω for branches with ω > 1.2	1.81	1.80	1.95	1.64	1.79
Mean ω ± SE	0.51 ± 0.44	0.62 ± 0.47	0.84 ± 0.65	0.28 ± 0.17	0.29 ± 0.17

^a^of analysed branches.

### Effect of cluster size and length

The UP clusters are longer and contain more sequences than the SO clusters (see above). This could lead to an underestimation of codons under selection in SO clusters as codeml has more power to detect footprints of positive selection in longer/larger alignments [39]. A general linear model analysis showed that differences in alignment length cannot explain the detected differences between UP and SO clusters if cluster size (=number of sequences in alignment) is ≤ 10 (data not shown). Cluster size, however, had an effect. In order to test the reliability of our results relative to the number of sequences, we performed Fisher's exact tests to see if we could find either significantly more clusters, codons, and/or branches under selection for UP than for SO cluster in each cluster size category (up to 10 as this is the maximum for SO clusters). We find significantly more clusters under positive selection for UP clusters for the size categories 6 and 7 (Table 4). For the other size categories we lack power to detect significant differences (Table 4). We also detect significantly more codons showing footprints of selection in UP clusters for the size categories 6–9 (Table 4). In addition, branches with ω > 1.2 are significantly more frequent in UP clusters for size categories 5–10 (Table 4). To summarize, UP clusters still show more signatures of positive selection more frequently after controlling for cluster size effect.

**Table 4:** Results of Fisher’s exact test

	**Number of clusters**			**Number of codons**			**Number of branches**		
**Cluster size**	**UP under/not under positive selection**	**SO under/not under positive selection**	***p*-value Fisher’s exact test**^**a**^	**UP under/not under positive selection**	**SO under/not under positive selection**	***p*-value Fisher’s exact test**^**a**^	**UP under/not under positive selection**	**SO under/not under positive selection**	***p*-value Fisher’s exact test**^**a**^
4	1/48	0/3	1	2/22,821	0/1,467	1	9/210	0/15	1
5	4/102	0/12	1	16/51,017	0/4,187	0.62	51/483	0/84	8.78E-04^*^
6	24/474	0/487	9.27E-08^***^	66/210,761	0/191,533	2.20E-16^***^	184/3,456	6/4,329	2.20E-16^***^
7	15/280	0/405	1.90E-06^***^	43/127,403	0/163,947	3.59E-16^***^	136/2,299	8/4,378	2.20E-16^***^
8	4/178	0/293	0.02	24/81,803	0/110,494	1.24E-09^***^	117/1,430	5/3,744	2.20E-16^***^
9	7/108	0/144	3.07E-03	19/49,429	0/57,346	4.42E-07^***^	69/1,110	7/2,085	2.20E-16^***^
10	4/73	0/26	0.57	14/36,324	0/10,298	0.05	76/714	1/425	6.03E-14^***^

The table contains the results of Fisher’s exact test for number of clusters, codons, and branches under positive selection vs. not under positive selection in UP and SO clusters for different cluster size categories.

^a^Bonferroni corrected for multiple testing.

^*^p < 2.38E-03, ^***^p < 4.76E-05.

### Effect of evolutionary time and polymorphism

**Figure 5:**
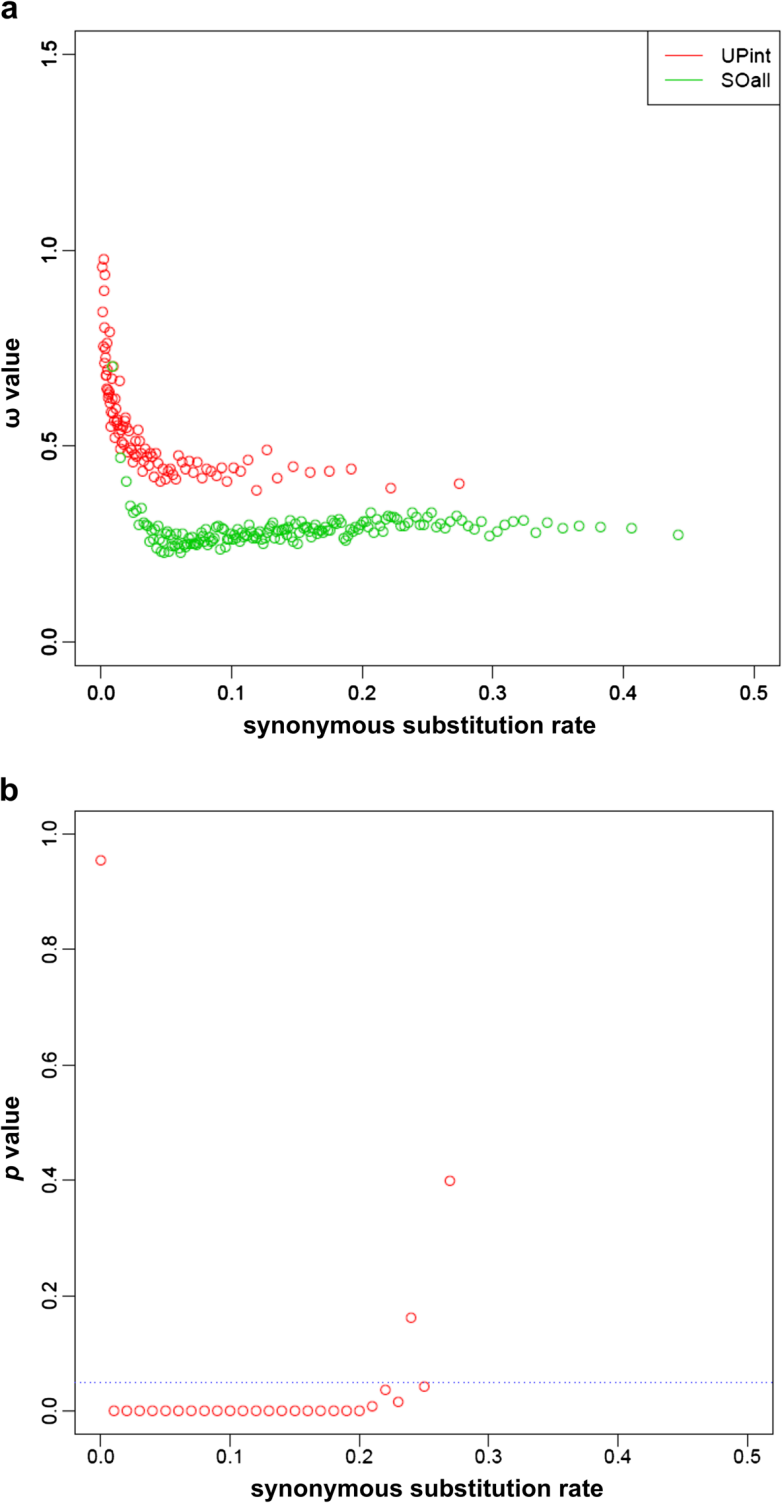
**ω of branches according to the ratio of synonymous mutations. (a)** ω of internal branches of UP clusters (red) and all branches of SO clusters (green) is plotted against the rate of synonymous mutations of sequences. As the point density is too high, each point represents the mean of 100 values. **(b)** The *p*-value of the Mann–Whitney test according to the synonymous substitution rate. This statistical test is performed using all the ω data and on intervals of 0.01 and contains at least 25 values. The dotted blue line is the significance level fixed at 0.05.

To see if our results are biased by divergence discrepancies between UP and SO, we sorted the ω value of each branch by their synonymous substitution rate (*d*_S_). To rule out the effect of polymorphism, we excluded (“young”) external branches from the dataset and compared the remaining (“old”) internal branches (UPint) to the SO dataset. We found a significant difference between the ω of SO and UPint in *d*_S_ intervals ranging from 0.01 to 0.21 (Figure 5a+b). There is no significant difference in the first *d*_S_ interval (Figure 5a), most likely because of residual polymorphism and/or a low mutation rate in SO and UP clusters. This interval harbors, however, more than 50% of the dataset. These results indicate that – except for very low *d*_S_ values – the difference between SO and UP cluster cannot be explained solely by divergence discrepancies or residual polymorphism. Above *d*_S_ values of 0.21 the Mann–Whitney test is inconclusive (Figure 5b) due to lack of power.

### Annotation of clusters under selection

The GreenPhylDB provides details on predicted molecular function, biological process, cellular component, and family and domain annotation for each cluster. We extracted those details for clusters found to have evolved under positive selection using codeml’s site model. Additional file 2 provides the details of the annotations for all the clusters with codons under selection before excluding clusters which derive from the same GreenPhyl tree (see Methods). Annotation is an ongoing process on the GreenPhylDB; therefore most of the clusters are not annotated – especially in monocots. There seems to be no trend in tree size or species specificity as clusters shown to have codons under selection can both be found in large trees containing sequences from various plant species and from small species specific trees (Additional file 2). As annotation is ongoing and remains under constant modification, a comprehensive analysis of the potential function of the clusters with codons under selection would not lead to reliable results. However, some trends can be observed: (i) the most abundant molecular function is “protein binding” (21.57% of all annotated molecular functions in the dataset) followed by “transferase activity” (9.80%). This is especially true in the Level 2 dataset (*i.e.* clusters derived from large GreenPhyl trees) whereas potential molecular functions seem to be more diverse in the Level 1 dataset (Additional file 2). (ii) The most common predicted biological functions are “metabolic process” (23.53% of all annotated biological processes in the dataset) and “oxidation-reduction process” (20.59%). “Defense” (14.71%) is also dominant, but only in the Level 2 dataset (Additional file 2). (iii) If domains are annotated to the clusters with codons under selection, F-box (22.54% of all annotated domains in the dataset), Leucine rich repeats (LRR; 11.27%), and NB-ARCs (8.45%) are predominant. Again, this trend is mostly visible in the Level 2 dataset whereas potential domains are more diverse in the Level 1 dataset (Additional file 2).

## Discussion

The important role of duplicated genes in plant adaptation has been argued theoretically (reviewed by [43]). To assess whether lineage specific expanded (LSE) genes show more evidence for positive selection than single-copy genes we analyzed LSE gene families from ten angiosperm genomes using a *d*_N_/*d*_S_-based test. We found positive selection footprints moderately frequently at the codon level in LSE genes (5.38% in average among the different species) but did not find any positive selection footprints on single-copy genes after manual curation. The number of codons under positive selection is also found higher in LSE than in single copy genes for different cluster size categories and thus cannot be explained solely by a difference of power to detect positive selection between the two datasets. Positive selection is also detected in LSE genes at the branch level and we found a significantly higher proportion of branches under positive selection among LSE gene trees than among single-copy ones. Inferring *d*_N_/*d*_S_ at the branch level is complementary to analyzing *d*_N_/*d*_S_ at the codon level. Using site models, *d*_N_/*d*_S_-based tests have the greatest power to detect footprints of selection in genes involved in co-evolutionary processes as a limited subset of their codons is repeatedly subject to positive selection (reviewed by [44]). At the branch level, the evolutionary rate is averaged over the complete amino acid sequence, making it difficult to detect a signal when only few sites are targets of positive selection. However, an elevated evolutionary rate can be detected even if it affects only certain lineages. When *d*_N_/*d*_S_ was computed on all the branches of the same dataset as for site analyses, we detected a stronger effect of positive selection on LSE genes compared to single-copy genes. Therefore, we argue that LSE genes are a much more important substrate for positive selection to act on than single-copy genes. This is – to our knowledge – the first genome-scale study to empirically demonstrate that LSE genes fuel adaptation in angiosperms.

Among the vast literature dealing with population genetic models of duplicated gene evolution, a crucial point is whether natural selection plays a role in it [18]. Positive selection is expected to act either on the fixation process of the duplication itself or at new mutations occurring after fixation of the copy in the species (or at both levels successively). We found a significantly larger portion of LSE genes under positive selection compared to single copy ones. Hence, the differentiation between copies for LSE genes is driven by changes in proteins, with all the functional consequences this may imply. This result corresponds to predictions made by several models, *e.g.* the “adaptation” model [16,19] or the “adaptive conflict” model [20-22]. In these scenarios, the duplication itself is not subject to positive selection, and may be fixed by genetic drift. However, our results may be coherent with a third scenario of segregation avoidance [45] where several alleles are pre-existing at the ancestral unique locus and their retention is advantageous [46,47]. Thus, duplications may favor the retention of those alleles if each of them gets fixed at one of the different locus resulting from the duplication process. In this scenario, positive selection does occur on the fixation process itself and the non-synonymous mutation observed would have appeared before the duplication process. However, it is not possible to tell which of these scenarios is more likely in our data, all the more that those scenarios can be combined in more complex ones. For instance, a first duplication may occur allowing a unique gene to escape an adaptive conflict and subsequent duplications may occur; generating additional copies following – this time – an adaptive scenario.

Recent progress in angiosperm whole genome sequencing gave numerous arguments in favor of the positive role of polyploidy in the exceptional radiation and diversification of angiosperms [48-50]. These hypotheses rely on the evolutionary potential caused by genomic shocks such as polyploidy. Our study shows that genomic events leading to gene duplications at a smaller scale – especially when recurring at a high frequency as it has been described in angiosperm genomes [8-10] – appear also fundamental in the adaptive dynamic of angiosperms. Recurrent gene duplication/retention offer a mechanism complementary to WGD as it may take place all along the evolutionary time and can affect a specific subset of gene families. Such families might be targeted according to their implication in biological processes or molecular functions related to the ongoing natural selective pressure. This could be reflected by the trends we observed in the annotations of the genes containing codons under selection: many are involved in defense and protein binding is the most common molecular function.

The most abundant domains we found in LSE clusters showing signatures of positive selection are F-box and LRR domains. F-box proteins (FBP) are one of the largest and fastest evolving gene families in land plants [51]. When analyzing FBP subfamilies in seven land plant species, it was found that 64-67% of duplications are species-specific – mostly in angiosperms [52,53]. Expression analysis of LSE FBPs showed a fast subfunctionalization on the transcriptional level [52,53]. Finally, it was also found that the LSE FBP are less conserved than their single-copy counterparts and signatures of positive selection are predominantly found in the protein-protein interaction domains of the FBPs [52,53]. An equally large gene family comprises of receptor-like kinases (RLK) containing LRRs in their extracellular domain [54]. Two main functions are described for LRR-RLKs: development and defense [55]. LRR-RLKs involved in defense are predominantly found in LSE gene clusters whereas LRR-RLKs involved in development are mostly found in non-expanded groups [55]. It was also discovered that the LRR domains are significantly less conserved than the remaining domains of the LRR-RLK genes [55]. In addition, a study on four plant genomes showed that LRR-RLK genes from LSE gene clusters show significantly more indication of positive selection or relaxed constraint than LRR-RLKs from non-expanded groups [55]. Therefore, it is not surprising that F-box and LRR domains are the most abundant domains we found in the LSE clusters with codons under positive selection. First, proteins containing these domains constitute large gene families and are therefore likely to show up in our LSE dataset – especially when coming from the GreenPhyl Level 2 dataset as it comprises of large trees. Second, several studies showed that these proteins/domains are prone to fast evolution and adaptation [51,55]. The results shown here give valuable insight in the evolution of large gene families and provide the groundwork for more detailed analyses of these candidates.

As automated multi-step genome wide analyses can sometimes introduce biases and misinterpretations, we took the maximum of precautions at each step. First, we chose well-annotated genomes to reduce the bias of mis-annotations, although we cannot completely rule them out. Annotation errors could lead to an over-estimation of the evolutionary rate in duplicated genes [56]. This left us with ten angiosperm genomes, even though many completely sequenced genomes are now available. Second, as *d*_N_/*d*_S_-based methods are very sensitive to alignment errors [57,58], reliable alignment and cleaning tools are mandatory. We used PRANK and GUIDANCE to align and clean the sequence clusters. Those recent methods have been found to produce the most reliable alignments for downstream analysis using the PAML software [57,58]. Third, we curated the alignments for which we detected positive selection manually. As this is a great deal of work in large datasets many studies fail to do this. However, we argue that this step is crucial to produce reliable results as we found around 50% alignment errors and therefore false positives. The manual validation of all the positively selected sites is a major strength of our study. Fourth, the power for *d*_N_/*d*_S_ analysis is related to the number of sequences aligned. In our dataset the difference in sequence number was significant between the LSE and the single-copy dataset. This could explain, at least partially, the detection of a higher number of clusters with sites under positive selection. By analyzing LSE and single-copy gene clusters in each size categories separately we ruled out the effect of cluster size and showed that the number of clusters, codons and branches under positive selection is always higher in LSE genes compared to single-copy genes. Fifth, we wanted control for a potential “family effect” that could result from the fact that some gene families showing accelerated evolutionary rate in general, *e.g.* because of their function or base composition, may also be more prone to gene duplication and/or retention than others. Using subgroups we indeed found an effect: LSE clusters from trees containing also a single-copy gene clusters show a lower *d*_N_/*d*_S_ compared to LSE clusters from trees without single-copy gene clusters. This means that the more a gene family is prone to duplication/retention the less probable a single-copy gene cluster will be found. Here, we give an argument in favor of the hypothesis that the initial level of selective constraint partially conditions the frequency of duplication/retention. We detect a family effect in different trees but the *d*_N_/*d*_S_ difference between LSE clusters and single-copy gene sets remains significant when controlling for this effect by comparing clusters extracted from the same gene trees.

Finally, when analyzing very recent duplicates it is possible that the differences between copies are still segregating within populations which violates basic assumptions of *d*_N_/*d*_S_-based tests [59]. Our LSE dataset may include genes where differences are still polymorphic which can lead to an overestimation of positive selection [59,60]. As expected, *d*_N_/*d*_S_ is elevated – and most likely over-estimated – for low *d*_S_ values in LSE as well as in single-copy gene clusters. The reason for this effect is either polymorphism segregating in young copies (mostly the case in LSE genes) or a low mutation rate (mostly the case in single-copy genes). However, even after removing external (“young”) LSE branches, the difference between single-copy and LSE gene clusters is still significant for *d*_S_ values above 0.01. This result shows that polymorphism and/or a low mutation rate alone cannot explain the differences in *d*_N_/*d*_S_ between LSE and single-copy genes.

Functional analysis is difficult in recently expanded gene families because functional or gene expression differences are difficult to investigate due to highly similar sequences among copies. Additionally, many of these genes are involved in stress responses [11,12] and therefore specific conditions need to be defined *a priori*. Consequently, molecular evolution studies like ours are a good alternative to identify candidates in which family expansion is followed by an adaptive process to conduct further analyses. Another next step could be to investigate links between our results and the duplication mode. By looking at the location of duplicated genes in the genome the duplication mode can be assessed. Several studies showed that the duplication mode has an impact on genetic novelty and adaptation [61,62]. For example, it was demonstrated that TDGs are more often involved in abiotic stress response than non-TDGs [10,11,63]. However, a *d*_N_/*d*_S_ approach is not suitable to provide evidence for positive selection on the duplication process itself which is the assumption under the dosage effect hypothesis [13]. Therefore, we ignore gene conservation as potential outcome and subsequently probably underestimate the role of adaptation in gene duplication/retention.

## Conclusions

In this paper we conduct one of the largest studies on the role of recurrent gene duplication on adaptation in angiosperms so far. Indeed, most of the former studies either dealt with candidate families in a broad taxonomical range (*e.g.*[35-37]) or whole genomes for a maximum of four plant species (*e.g.*[11,12]). We searched duplicated genes from ten angiosperm genomes for footprints of positive selection and our results provide candidates for further functional or population genetic studies. In general, we used a very conservative approach to detect positive selection footprints at LSE genes and might therefore miss many true positives. Still, because of the inherent differences between LSE and single-copy datasets, our results must be interpreted with caution. As the number and quality of sequenced genomes is increasing daily, our analysis can be expanded to many more plant species in the future. In addition, current efforts in re-sequencing numerous genomes from different populations could give the opportunity to differentiate between divergence and polymorphism and to consequently provide even better estimates of quantity and quality of positive selection undergone by LSE genes.

## Methods

### Genomes, proteomes, identification of ultraparalog clusters and superortholog gene sets

As analysis of duplicated genes is very sensitive to gene annotation errors we chose five well annotated monocot and five well annotated dicot genomes (see details on our genome selection criteria in Additional file 1): *Musa acuminata* v1.0 (banana) [5], *Oryza sativa* subsp. *japonica* v6.0 TEfiltered (Asian rice) [9], *Brachypodium distachyon* v1.0 (purple false brome) [64], *Zea mays* v5.6 filtered (maize) [65], *Sorghum bicolor* v1.4 (milo) [66], *Vitis vinifera* v1.0 (common grape vine) [4], *Arabidopsis thaliana* v10.0 (thale cress) [8], *Populus trichocarpa* v2.2 (black cottonwood) [67], *Glycine max* v1.0 (soybean) [68], and *Medicago truncatula* v3.5 (barrel medic) [69]. The phylogeny of those species is provided in Additional file 1. We used the information provided by the GreenPhyl v3 database (http://www.greenphyl.org) which uses a tree reconciliation approach [70] to identify orthologs (genes related by speciation) and paralogs (genes related by duplication) in protein trees. This database contains protein families’ composition and phylogenies for a broad variety of green plants whose genomes have been completely sequenced [41]. Based on their sequence similarity, the GreenPhylDB clusters gene families at different levels from the less stringent (large clusters of relatively similar sequences at Level 1) to the most stringent (small clusters of highly similar sequence at Level 4). First, we extracted 3,330 protein clusters from Level 1. As large gene families (>500 sequences) are not further analyzed in GreenPhyl, we extracted 2,238 protein clusters from Level 2 for these gene familie. These are two separate datasets and Level 2 trees are not nested in Level 1 tress (see GreenPhyl homepage for details: http://www.greenphyl.org/).

We extracted ultraparalog clusters (UP – sequences only related by duplication) from the GreenPhylDB trees on which duplication and speciation events were positioned according to the tree reconciliation approach cited previously (Figure 1a). Those clusters represent our LSE gene set. As a single-copy gene reference, we chose a superortholog gene set (SO – sequences only related by speciation). We ignored clusters with less than six sequences. The SO clusters were divided into clusters coming from the same tree as UP clusters (SO1) or from trees exclusively harboring SO clusters (SO2). Likewise, UP clusters were divided in clusters coming from trees containing SO clusters (UP1) or from trees with only UP clusters (UP2). Note that when a GreenPhyl tree harbors several SO and/or UP clusters, all were extracted. We downloaded the corresponding complete CDS of the species of interest (links on GreenPhylDB Documentation section). In case of alternative spliceforms, the longest one is kept in the GreenPhylDB pipeline; it is thus the one we downloaded. Most GreenPhyl trees are too large and/or too divergent to create reliable nucleotide alignments and perform *d*_N_/*d*_S_-based tests on the whole tree alignment. This is especially true for the most interesting cases where trees contain both UP and SO clusters (the UP1/SO1 dataset). We therefore chose to analyze each UP and SO cluster independently.

In GreenPhyl trees harboring several UP and/or SO clusters *i.e.* in gene families in which gene duplication/retention might be more frequent, one might expect selective constraint to be different, in particular more relaxed. Therefore, some gene families might be overrepresented when several clusters from the same tree are analyzed separately. To avoid this, an additional step of selection was added to the initial dataset as we randomly kept only one cluster each time several clusters of UP or several clusters of SO were identified from a same tree and removed all other clusters from our analysis. Here, we present the results for this final sub-dataset. However, we performed our analysis on three additional sub-datasets: (i) the whole dataset without removing clusters from trees harboring more than one cluster, (ii) a dataset which contains clusters from GreenPhyl trees with only one UP and/or one SO cluster, (iii) a dataset where only clusters from trees harboring more than one cluster were kept. The results for these sub-datasets can be found in Additional file 1. However, the trends we observe remain, no matter which sub-dataset is analyzed (Additional file 1).

### Alignment and cleaning

We used PRANK_+F_ with codon option [71] for creating the alignments and GUIDANCE [42] with the default sequence quality cut-off and a column cut-off of 0.97 to remove problematic sequences and unreliable sites from the initial alignments (Figure 1b). Those choices were guided by several recent studies which found PRANK_codon_ and the PRANK_codon_-GUIDANCE combination to produce the most reliable alignments for further inference of positive selection using codeml [57,58]. Filtering removed all sequences from 33 UP clusters, it kept three or less sequences for 91 UP and two SO clusters; all those clusters were thus ignored in further analyses as a minimum of four sequences was required. For some species (namely *Z. mays*, *S. bicolor*, *G. max*, and *M. truncatula*), the retrieved CDS seemed to contain un-translated regions (UTRs) as for 126 UP and four SO clusters one or more sequences contained stop codons or incomplete codons (*i.e.* length not divisible by three). Those clusters were also removed from the analysis. Additionally, for 18 UP clusters codeml failed to run (probably due to insufficient sequences overlap). We retrieved 167 UP1 and 167 SO1 as well as 1,656 UP2 and 1,203 SO2 clusters. After cleaning, our final dataset for codeml analysis comprised 160 UP1, 1,512 UP2, 167 SO1, and 1,203 SO2 clusters for the codeml analysis (Table 1).

As alignment errors can create false positives in the detection of positive selection footprints, each cluster suggested to be under positive selection was again checked both automatically – using muscle [72] and trimAL [73] for creating and cleaning alignments (muscle-trimAL method; see Additional file 1) – and manually for alignment errors. We found that our initial alignment and cleaning procedure using PRANK [71] and GUIDANCE [42] is superior to the muscle-trimAL method. Manual curation, however, remains essential to avoid false positives (Additional file 1).

### Detecting codons under positive selection

We used codeml site model implemented in the PAML4 software [74] to infer positive selection on codons under several substitution models. For these analyses, we extensively relied on the egglib package [75] to implement the following pipeline: First, for every alignment the maximum likelihood phylogeny was inferred at the nucleotide level using PhyML 3.0 [76] under the GTR-Γ substitution model (Figure 1c). Second, different codeml site models were run (Figure 1d). The nearly neutral models (M1a and M8a) assume codons to evolve either neutrally or under purifying selection whereas the positive selection models (M2a and M8) assume positive selection acting on some codons. Third, likelihood ratio tests (LRTs) were performed using R [77] to compare nearly neutral and positive selection models and hence to detect clusters for which models including positive selection are significantly more likely than models that do not. We corrected for multiple testing using a Bonferroni correction. In clusters identified to have evolved under positive selection, Bayes empirical Bayes was used to calculate the posterior probabilities at each codon and detect those under positive selection (*i.e.* those with a posterior probability of ω > 1 strictly above 95%). All alignments detected to be under positive selection at the codon level were curated manually for potential alignment errors. More details on the estimated omega for each cluster with codons under positive selection, position of every codon under positive selection, and results of the LRT for those clusters can be found in Additional file 3. All cleaned alignments containing codons under positive selection are provided in Additional file 4.

### Assessing d_*N*_/d_*S*_ at branches

For inferring ω on branches, the alignments and the corresponding phylogenies were used as input for mapNH [78,79]. Unlike the branch-site model in codeml, this method does not require to define branches under selection *a priori*[78]. mapNH performs substitution mapping before clustering branches according to their underlying substitution processes (Figure 1d). The ω of each branch was then calculated as followed:

$$\omega =\begin{array}{c} \mathrm{nbNS}/\mathrm{NSsites}\\ \mathrm{nbS}/\mathrm{Ssites} \end{array}$$

using *nbNS* (number of non-synonymous mutations) and *nbS* (number of synonymous mutations) estimations provided by mapNH whereas *NSsites* (number of non-synonymous sites) and *Ssites* (number of synonymous sites) were computed by codeml during the site model analysis. We preferably used the *NSsites* and *Ssites* provided by codeml since they benefit from the maximum likelihood estimation of the transition/transversion ratio done by codeml for each alignment. Finally, note that ω was estimated only for clusters with at least one synonymous and one non-synonymous mutation. After clusters with no mutation were removed for the mapNH analysis, 154 UP1, 1,435 UP2, 167 SO1, and 1,203 SO2 clusters remained (Table 1). Branches containing no substitutions were also removed, leaving us with 1,257 UP1, 14,326 UP2, 1,807 SO1, and 13,374 SO2 branches for the final analysis (Table 1).

### Determining effects of time and polymorphism

SO and UP clusters are different by definition. First, the divergence times between sequences are not expected to be the same. Specifically, divergence in a given SO cluster should range between minimum and maximum divergence time of the species included in this cluster. Divergence in UP clusters should range from null (for very recent duplications) to the last speciation event. It has been shown that *d*_N_/*d*_S_-based tests are strongly influenced by *d*_S_[59]. To test whether our results are biased due to divergence discrepancies between UP and SO, we sorted the ω value of each branch by their synonymous substitution rate (*d*_S_). Second, in the UP dataset some duplications could have occurred very recently. It is likely that some differences between those young paralogs are still segregating in populations and should therefore be considered as polymorphism instead of divergence. Inferring selection using *d*_N_/*d*_S_ in such a scenario has been shown to be incorrect [60]. To rule out effects of polymorphism on UP clusters, we excluded external branches from the dataset and compared the remaining internal branches to the SO dataset. To test if ω differs significantly between types of clusters, we performed a Mann–Whitney test using R [77]. When ω is analyzed according to *d*_S_, Mann–Whitney tests were performed in a sliding window of 0.01 *d*_S_. The calculation was done when a window contained at least 100 values by group studied.

## Availability of supporting data

The data sets supporting the results of this article are included within the article and its additional files.

## Abbreviations

WGD: Whole genome duplication; SSD: Short scale genomic duplication; TDG: Tandemly duplicated gene; LSE: Lineage specific expansion; UP: Ultraparalog; SO: Superortholog; LRR: Leucine-rich repeat; RLK: Receptor-like kinase; FBP: F-box protein.

## Competing interests

The authors declare that they have no competing interests.

## Authors’ contribution

IF, JD, VR, JFD, and NC designed the study; IF, JD, and JFD developed the pipeline; IF, JD, SG, and JFD performed the data analysis and statistics; IF drafted the manuscript with the help of JD, VR, SG, JFD, and NC. All authors read and approved of the final manuscript.

## Supplementary Material

Additional file 1 **Extended Materials and Methods and extended Results.** This includes: **Table S1:** Clusters initially under selection and number and percentage of clusters removed after manual inspection and after applying the muscle-trimAL pipeline for Level 1 the dataset. **Table S2:** Clusters containing codons under positive selection according to the codeml site model before and after manual curation in the whole dataset. **Table S3:** Clusters containing codons under positive selection according to the codeml site model before and after manual curation in the dataset containing clusters from GreenPhyl trees with only one UP and/or SO cluster. **Table S4:** Clusters containing codons under positive selection according to the codeml site model before and after manual curation in the dataset containing only clusters from trees harboring several clusters. **Table S5:** Results of the mapNH analysis for the different datasets. **Figure S1:** Phylogeny of a subset of plant species of the GreenPhylDB. **Figure S2:** Overview of the different sub-datasets analyzed. Click here for file

Additional file 2 Excel spreadsheet containing GreenPhyl gene families found to be under positive selection with the codeml site model. Click here for file

Additional file 3 Excel spreadsheet that contains the estimated omega for each cluster with codons under selection, results of the LRT for those clusters, and the position of every manually curated codon under positive selection in the provided alignments. Click here for file

Additional file 4 Contains the cleaned alignments of the clusters with codons under selection. Click here for file
