# Supplementary material for: Impact of recurrent gene duplication on adaptation of plant genomes
Source: BMC Plant Biol. 2014 May 31;14:151. doi: 10.1186/1471-2229-14-151 (PMC4049390; doi:10.1186/1471-2229-14-151)
Supplement: Additional file 1 — Extended Materials and Methods and extended Results. This includes: Table S1: Clusters initially under selection and number and percentage of clusters removed after manual inspection and after applying the muscle-trimAL pipeline for Level 1 the dataset. Table S2: Clusters containing codons under positive selection according to the codeml site model before and after manual curation in the whole dataset. Table S3: Clusters containing codons under positive selection according to the codeml site model before and after manual curation in the dataset containing clusters from GreenPhyl trees with only one UP and/or SO cluster. Table S4: Clusters containing codons under positive selection according to the codeml site model before and after manual curation in the dataset containing only clusters from trees harboring several clusters. Table S5: Results of the mapNH analysis for the different datasets. Figure S1: Phylogeny of a subset of plant species of the GreenPhylDB. Figure S2: Overview of the different sub-datasets analyzed. [file 1471-2229-14-151-S1.pdf]

Supplementary Online Material – File 1

Fischer et al.

Impact of recurrent gene duplication on adaptation of plant genomes

<u>Extended Materials and Methods</u>

<u>Plant Genomes</u>

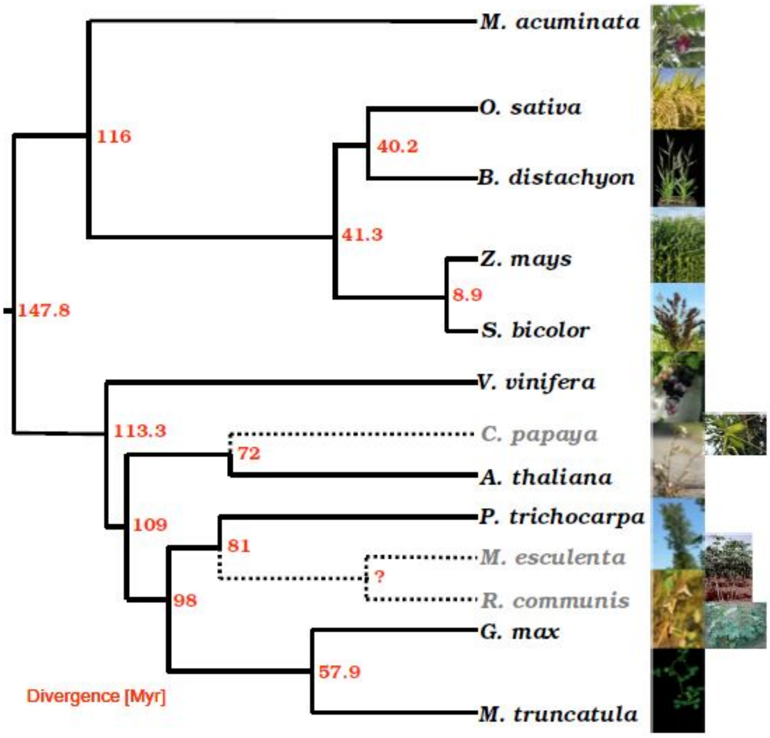

Figure S1: Phylogeny of a subset of plant species of the GreenPhylDB. Dashed lines indicate species which were not used in our analysis. Numbers at nodes show the divergence time in Myr (million years). Data on divergence time "expert results" from: http://www.timetree.org/; 26.06. 2013. Pictures from Wikipedia.org.

As analysis of gene families is very prone to annotation errors we chose to focus our analysis on genomes that: (i) were sequenced, at least partially, with a Sanger-related sequencing approach, (ii) have a coverage around 10x or higher, (iii) have > 90% of their coding genes covered, (iv) have the protein coding genes annotation checked manually, (v) were linked to a genetic/physical/optic map, and (vi) are not the first version. As fulfilling all criteria would have led us with a dataset too small for a comprehensive analysis, we chose a "top ten" of genomes fulfilling most criteria. The phylogeny of the species we used is provided in Figure S1. Using the information on plant proteomes available on the GreenPhyl database [1], we

identified ultraparalog (UP – only related by duplication) clusters and superortholog (SO – only related by speciation) gene sets and extracted the corresponding CDS for further analysis. Based on their sequence similarity, the GreenPhylDB clusters gene families at different levels from the less stringent (large clusters of relatively similar sequences at Level 1) to the most stringent (small clusters of highly similar sequence at Level 4). We concentrated on the trees from Level 1 and Level 2.

Alignment and Cleaning

We used $\text{PRANK}_{\text{codon}}$ [2] and GUIDANCE [3] to align and clean the UP and SO clusters as $\text{PRANK}_{\text{codon}}$ and the $\text{PRANK}_{\text{codon}}$-GUIDANCE combination were found to produce the most reliable alignments for further inference of positive selection using codeml [4, 5]. As alignment errors can create false positives in the codeml analysis, each cluster suggested to be under positive selection was manually checked for alignment errors. This method is rather time consuming and we therefore re-run the pipeline with another alignment and cleaning method (muscle-trimAL) on the clusters identified to be under selection. It allowed us to determine if the results of codeml are consistent and if not, which alignment is more reliable. We performed the alignments with the muscle software [6] wrapper in egglib [7]. muscle performed third best after $\text{PRANK}_{\text{codon}}$ and PRANK_{aa} [2] in benchmarks to assess alignment certainty for further codeml analysis [4, 5]. In order to keep the phase of our codon alignments, we ran muscle on translated alignments and transferred it back to coding sequences using the ‘translate’ and ‘backalign’ tools available in egglib. Cleaning was performed using trimAL [8], an alignment trimming tool. We used the ‘gappyout’ option which automatically calculates the most appropriate gap cut-off point and removes all columns not reaching this threshold. To remove poorly aligned sequences, we set the residue overlap threshold to 0.65 and the sequence overlap threshold to 50.

In most cases the results of $\text{PRANK}_{\text{codon}}$-GUIDANCE [3] and muscle-trimAL were consistent, but the muscle-trimAL alignment tended to produce more extreme *p*-values in the likelihood ratio tests (data not shown). Comparison of both alignments showed that this was mostly due to mis-alignments by the muscle-trimAL pipeline which were absent in the $\text{PRANK}_{\text{codon}}$-GUIDANCE alignments. However, almost 27% of the muscle-trimAL alignments were not identified to be under selection anymore, with *A. thaliana* showing

relatively few (9.09%) and *V. vinifera* showing many (45.45%) such cases (Table S1). There are two possible reasons for that. First, the sequences in the clusters were so divergent that trimAL removed all of them whereas $PRANK_{codon}$ and GUIDANCE aligned them by introducing many gaps. However, all of these alignments were clearly mis-aligned and would have been removed by manual curation. Second, the models for selection (M2a and M8) performed not significantly better than the neutral models (M1a and M8a) anymore. Here, two scenarios were responsible for that. In the first scenario, only a small part was well aligned and the rest was cut by trimAL whereas $PRANK_{codon}$-GUIDANCE kept those parts by introducing gaps and creating false positives. Again, these cases could be clearly identified and removed by manual curation. In the second scenario, GUIDANCE demonstrated its power to maintain true positives [5] which were rigorously cut by trimAL. These alignments were kept after manual curation. In the Level 1 dataset, 46.04% of the alignments were removed by manual curation (Table S1). As manual curation showed to be more efficient and conservative than the automated muscle-trimAL strategy, we decided to only keep alignments passing manual inspection as true positives. We therefore did not run the muscle-trimAL pipeline again on the Level 2 dataset. Here, 55.73% of the alignments were removed by manual curation (data not shown).

Table S1: Clusters initially under selection and number and percentage of clusters removed after manual inspection and after applying the muscle-trimAL pipeline for Level 1 the dataset.

Species	Clusters under selection - PRANKcodon-GUIDANCE alignments	Clusters no more under selection - muscle-trimAL alignments (%)	Clusters removed after manual inspection (%)
Musa acuminata	3	0 (0.00)	2 (66.67)
Oryza sativa	23	6 (26.09)	10 (43.48)
Brachypodium distachyon	10	3 (30.00)	9 (90.00)
Zea mays	18	5 (27.78)	8 (44.44)
Sorghum bicolor	10	2 (20.00)	5 (50.00)
Vitis vinifera	11	5 (45.45)	7 (63.64)
Arabidopsis thaliana	11	1 (9.09)	4 (36.36)
Populus trichocarpa	13	3 (23.08)	3 (23.08)
Glycine max	5	1 (20.00)	1 (20.00)
Medicago truncatula	35	8 (22.86)	19 (54.29)
**Total**	**139**	**37 (26.62)**	**64 (46.04)**

Creating Sub-Datasets

In total, we found 2,781 UP and 1,559 SO clusters (Figure S2). In the whole dataset, 203 SO clusters come from trees which also contain at least one UP cluster (=SO1; Figure S2).

Likewise, 255 UP clusters come from trees that also harbor at least one SO1 cluster (= UP1; Figure S2). 2,526 UP clusters come from trees not containing any SO clusters, but might contain other UP clusters (=UP2; Figure S2). 1,356 SO clusters, on the other hand, come from trees not containing UP clusters, but might contain other SO clusters (=SO2; Figure S2).

For the dataset presented in the main text, we randomly kept one cluster which comes from trees containing more than one cluster and removed all other clusters from our analysis. This dataset allows for more conservative analysis. One might expect a family effect in trees which contain several UP or SO clusters as in those gene families selective constraint might already be relaxed and gene duplication/retention might be more/less frequent. Therefore, the frequency of positive selection might be overestimated when several clusters from the same tree are analyzed independently. To gain this dataset, 88 UP1, 870 UP2, 36 SO1, and 153 SO2 clusters were removed (Figure S2; see main text for more details).

Two more datasets were created to see if our result depend on the presence/absence multiple clusters from the same trees. For the third dataset, all clusters from trees containing more than one cluster were removed. This left us with 122 UP1, 1,273 UP2, 142 SO1, and 1,091 SO2 clusters (Figure S2). Finally, we created a dataset comprising only clusters from trees harboring more than one cluster. This dataset contains 133 UP1 clusters from 45 trees, 1,235 UP2 clusters from 383 trees, 61 SO1 clusters from 25 trees, and 265 SO1 clusters from 112 trees (Figure S2).

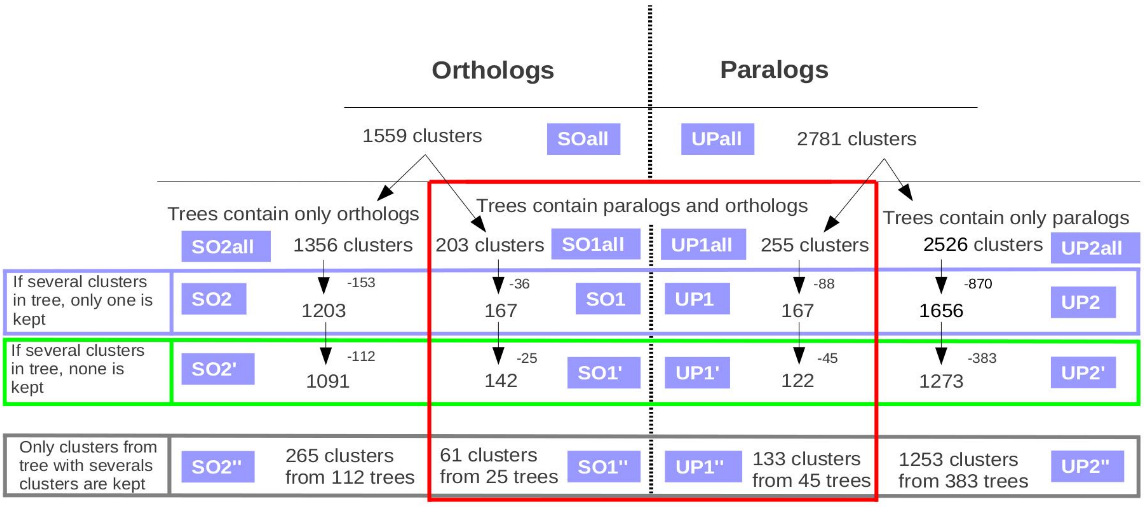

Figure S2: Overview of the different sub-datasets analyzed. The dashed line separates superortholog gene sets (SO = only related by speciation) and ultraparalog clusters (UP = only

related by duplication). UP1 and SO1 clusters come from the same GreenPhyl tree (red rectangle); UP2 and SO2 come from different GreenPhyl trees. One sub-dataset contains clusters for which only one cluster was randomly kept in case more than one of them came from the same GreenPhyl tree (light blue rectangle). A second subset contains clusters from GreenPhyl trees with only one UP and/or SO cluster (green rectangle). A third subset contains clusters for which only clusters from GreenPhyl trees with more than one of them (grey rectangle).

Extended Results

Codeml and mapNH Analysis

We ran the codeml site model [9] and mapNH [10, 11] on the different datasets described above. Tables S2-4 and Table 2 in the main text show the number and percentage of clusters for which positive selection was inferred at codons. Table S2 gives the results for the whole dataset; Table 2 in the main text gives the results for the dataset for which only one cluster was randomly kept in cases where several clusters come from the same GreenPhyl tree; Table S3 gives the results for the dataset which contains clusters from GreenPhyl trees with only one UP and/or SO cluster; Table S4 gives the results for the dataset where only clusters from GreenPhly trees which contain more than one cluster were kept. Finally, Table S5 contains the results of the mapNH analysis for all sub-sets. Although the results differ slightly between datasets, the main conclusion remains the same: we detect the same level of codons under selection and branches with $\omega > 1$ in UP and SO clusters.

Table S2: Clusters containing codons under positive selection according to the codeml site model before and after manual curation in the whole dataset.

	Clusters used in Final Analysis		Clusters under selection before manual curation (%)		Clusters under selection after manual curation (%)	
**Species**	**UP1**	**UP2**	**UP1**	**UP2**	**UP1**	**UP2**
M. acuminata	44	142	1 (2.27)	7 (4.93)	0 (0.00)	5 (3.52)
O. sativa	17	219	3 (17.65)	39 (17.81)	2 (11.76)	13 (5.94)
B. distachyon	4	94	0 (0.00)	16 (17.02)	0 (0.00)	3 (3.19)
Z. mays	33	326	5 (15.15)	49 (15.03)	0 (0.00)	14 (4.29)
S. bicolor	5	170	0 (0.00)	18 (10.59)	0 (0.00)	8 (4.71)
V. vinifera	19	189	2 (10.53)	19 (10.05)	0 (0.00)	10 (5.29)
A. thaliana	20	197	0 (0.00)	31 (15.74)	0 (0.00)	18 (9.14)
P. trichocarpa	29	235	3 (10.34)	48 (20.43)	1 (3.45)	38 (16.17)
G. max	26	219	3 (11.54)	14 (6.39)	3 (11.54)	5 (2.28)
M. truncatula	38	487	5 (13.16)	68 (13.96)	4 (10.53)	32 (6.57)
**Sum/Average**	**235**	**2,278**	**22 (9.36)**	**309 (13.56)**	**10 (4.26)**	**146 (6.41)**
**UPall**	**2,513**		**331 (13.17)**		**156 (6.21)**	
SO1	203		1 (0.49)		0 (0.00)	
SO2	1,356		4 (0.29)		0 (0.00)	

Table S3: Clusters containing codons under positive selection according to the codeml site model before and after manual curation in the dataset containing clusters from GreenPhyl trees with only one UP and/or SO cluster.

	Clusters used in Final Analysis		Clusters under selection before manual curation (%)		Clusters under selection after manual curation (%)	
**Species**	**UP1**	**UP2**	**UP1**	**UP2**	**UP1**	**UP2**
M. acuminata	27	71	0 (0.00)	2 (2.82)	0 (0.00)	1 (1.41)
O. sativa	3	106	0 (0.00)	20 (18.87)	0 (0.00)	7 (6.60)
B. distachyon	2	39	0 (0.00)	10 (25.64)	0 (0.00)	2 (5.13)
Z. mays	23	180	4 (17.39)	26 (14.44)	0 (0.00)	9 (5.00)
S. bicolor	2	66	0 (0.00)	5 (7.58)	0 (0.00)	3 (4.55)
V. vinifera	7	79	1 (14.29)	7 (8.86)	0 (0.00)	3 (3.80)
A. thaliana	11	116	0 (0.00)	25 (21.55)	0 (0.00)	14 (12.07)
P. trichocarpa	10	89	2 (20.00)	10 (11.24)	0 (0.00)	7 (7.87)
G. max	8	76	3 (37.50)	3 (3.95)	3 (37.5)	1 (1.32)
M. truncatula	22	310	3 (13.64)	41 (13.23)	3 (13.64)	18 (5.81)
**Sum/Average**	**115**	**1,132**	**13 (11.30)**	**149 (13.16)**	**6 (5.22)**	**65 (5.74)**
**UPall**	**1,247**		**162 (12.99)**		**71 (5.69)**	
SO1	142		1 (0.70)		0 (0.00)	
SO2	1,091		3 (0.27)		0 (0.00)	

Table S4: Clusters containing codons under positive selection according to the codeml site model before and after manual curation in the dataset containing only clusters from trees harboring several clusters.

	**Clusters used in Final Analysis**		**Clusters under selection before manual curation (%)**		**Clusters under selection after manual curation (%)**	
**Species**	**UP1**	**UP2**	**UP1**	**UP2**	**UP1**	**UP2**
M. acuminata	17	71	1 (5.88)	5 (7.04)	0 (0.00)	4 (5.63)
O. sativa	14	113	3 (21.43)	19 (16.81)	2 (14.29)	6 (5.31)
B. distachyon	2	55	0 (0.00)	6 (10.91)	0 (0.00)	1 (1.82)
Z. mays	10	146	1 (10.00)	23 (15.75)	0 (0.00)	5 (3.42)
S. bicolor	3	104	0 (0.00)	13 (12.50)	0 (0.00)	5 (4.81)
V. vinifera	12	110	1 (8.33)	12 (10.91)	0 (0.00)	7 (6.36)
A. thaliana	9	81	0 (0.00)	6 (7.41)	0 (0.00)	4 (4.94)
P. trichocarpa	19	146	1 (5.26)	38 (26.03)	1 (5.26)	31 (21.23)
G. max	18	143	0 (0.00)	11 (7.69)	0 (0.00)	4 (2.80)
M. truncatula	16	177	2 (12.5)	27 (15.25)	1 (6.25)	14 (7.91)
**Sum/Average**	**120**	**1,146**	**9 (7.50)**	**160 (13.96)**	**4 (3.33)**	**81 (7.07)**
**UPall**	**1,266**		**169 (13.01)**		**85 (6.71)**	
SO1	61		0 (0.00)		0 (0.00)	
SO2	265		1 (0.38)		0 (0.00)	

Table S5: Results of the mapNH analysis for the different datasets.

dataset	whole dataset				one cluster from trees containing several clusters randomly kept				all clusters from trees containing several clusters removed				only clusters from trees containing several clusters			
**subset**	**UP1**	**UP2**	**SO1**	**SO2**	**UP1**	**UP2**	**SO1**	**SO2**	**UP1**	**UP2**	**SO1**	**SO2**	**UP1**	**UP2**	**SO1**	**SO2**
Clusters for codeml analysis	235	2,278	203	1,356	160	1,512	167	1,203	115	1,132	142	1,091	120	1,146	61	265
Clusters for mapNH analysis	227	2,160	203	1,356	154	1,435	167	1,203	110	1,078	142	1,091	117	1,082	61	265
Studied branches/ Total number of branches	1,992/ 2,952	21,984/ 33,511	2,213/ 2,223	15,116/ 15,286	1,257/ 1,881	14,326/ 22,475	1,807/ 1,817	13,374/ 13,537	888/ 1,362	10,775/ 17,244	1,539/ 1,548	12,126/ 12,281	1,104/ 1,590	11,209/ 16,267	674/ 675	2,990/ 3,005
Branches with ω <1 (%)	1,846 (92.67%)	19,339 (87.97 %)	2,205 (99.64%)	15,069 (99.69%)	1,144 (91.01%)	12,515 (87.36 %)	1,799 (99.56%)	13,329 (99.66%)	802 (90.32%)	9,274 (86.07%)	1,5431(9 9.48%)	12,085 (99.66%)	1,044 (94.57%)	10,065 (89.79%)	674 (100 %)	2,984 (99.80%)
Mean ω for branches with ω	0.39	0.48	0.27	0.29	0.41	0.49	0.28	0.29	0.42	0.50	0.28	0.29	0.37	0.46	0.26	0.27
Branches with ω>1 (%)	188 (7.33%)	2,645 (12.03%)	8 (0.36%)	47 (0.31%)	113 (8.99%)	1,811 (12.64%)	8 (0.44%)	45 (0.34%)	86 (9.68%)	1,501 (13.93%)	8 (0.52%)	41 (0.34%)	60 (5.43%)	1,144 (10.21%)	0 (0%)	6 (0.20%)
Mean ω for branches with ω > 1	1.54	1.52	1.37	1.45	1.55	1.52	1.37	1.44	1.56	1.53	1.37	1.47	1.50	1.51	NA	1.30
Mean ω ± SD	0.48± 0.41	0.60± 0.46	0.28± 0.16	0.29± 0.17	0.51± 0.44	0.62± 0.47	0.28± 0.17	0.29± 0.17	0.53± 0.47	0.64± 0.49	0.29± 0.17	0.29± 0.17	0.44± 0.35	0.57± 0.43	0.26± 0.14	0.28± 0.15

References:

1. Rouard M, Guignon V, Aluome C, Laporte MA, Droc G, Walde C, Zmasek CM, Périn C, Conte MG: **GreenPhylDB v2.0: comparative and functional genomics in plants**. *Nucleic Acids Res* 2011, **39:**D1095-1102.
2. Löytynoja A, Goldman N: **An algorithm for progressive multiple alignment of sequences with insertions**. *Proc Natl Acad Sci USA* 2005, **102:**10557-10562.
3. Penn O, Privman E, Landan G, Graur D, Pupko T: **An alignment confidence score capturing robustness to guide tree uncertainty**. *Mol Biol Evol* 2010, **27:**1759-1767.
4. Fletcher W, Yang Z: **The effect of insertions, deletions, and alignment errors on the branch-site test of positive selection**. *Mol Biol Evol* 2010, **27:**2257-2267.
5. Jordan G, Goldman N: **The effects of alignment error and alignment filtering on the sitewise detection of positive selection**. *Mol Biol Evol* 2012, **29:**1125-1139.
6. Edgar RC: **MUSCLE: multiple sequence alignment with high accuracy and high throughput**. *Nucleic Acids Res* 2004, **32:**1792-1797.
7. De Mita S, Siol M: **EggLib: processing, analysis and simulation tools for population genetics and genomics**. *BMC Genet* 2012, **13:**27.
8. Capella-Gutiérrez S, Silla-Martínez JM, Gabaldón T: **trimAl: a tool for automated alignment trimming in large-scale phylogenetic analyses**. *Bioinformatics* 2009, **25:**1972-1973.
9. Yang Z: **PAML 4: phylogenetic analysis by maximum likelihood**. *Mol Biol Evol* 2007, **24:**1586-1591.
10. Dutheil JY, Galtier N, Romiguier J, Douzery EJ, Ranwez V, Boussau B: **Efficient selection of branch-specific models of sequence evolution**. *Mol Biol Evol* 2012, **29:**1861-1874.
11. Romiguier J, Figuet E, Galtier N, Douzery EJ, Boussau B, Dutheil JY, Ranwez V: **Fast and robust characterization of time-heterogeneous sequence evolutionary processes using substitution mapping**. *PLoS One* 2012, **7:**e33852.
